# Beyond Calories—Changes in Protein Supply Structure and Implications for Sustainable Food Systems in Sub-Saharan Africa

**DOI:** 10.3390/nu18142372

**Published:** 2026-07-20

**Authors:** Teodor Ioan Trasca, Ioana Mihaela Balan, Nicoleta Mateoc-Sirb, Aisha Simbiat Hussaini, Annie Gaise Magomboane, Sorin Mihai Cimpeanu

**Affiliations:** 1 Faculty of Animal Production Engineering and Management, University of Agronomic Sciences and Veterinary Medicine of Bucharest, 59 Marasti Blvd, District 1, 011464 Bucharest, Romania; teodor.trasca@usamv.ro; 2 Faculty of Management and Rural Tourism, University of Life Sciences “King Mihai I” from Timisoara, Calea Aradului 119, 300645 Timisoara, Romania; nicoletamateocsirb@usvt.ro; 3 Timisoara Branch, Research Center for Sustainable Rural Development of Romania, Romanian Academy, 010071 Bucharest, Romania; 4 Department of Agricultural Economic, Ahmadu Bello University, Samaru Campus, Community Market, 5M23+GR3, Zaria 810211, Kaduna, Nigeria; ashussaini@abu.edu.ng; 5 Faculty of Agriculture, University of Uele, 91 Avenue de Langhes, Isiro B.P. 670, Haut-Uele, Democratic Republic of the Congo; anniegaisse@gmail.com; 6 Faculty of Land Reclamation and Environmental Engineering, University of Agronomic Sciences and Veterinary Medicine of Bucharest, 59 Marasti Blvd, District 1, 011464 Bucharest, Romania; mscimpeanu@yahoo.fr

**Keywords:** food systems, protein supply structure, adjusted protein density, animal-based protein, plant-based protein, dietary quality, Sub-Saharan Africa, food system transformation, sustainable diets, SDG 2 Zero Hunger

## Abstract

**Background/Objectives:** Food system assessments in Sub-Saharan Africa have predominantly focused on caloric availability, often overlooking the structural and nutritional quality of the food supply. This study examines what drives and constrains food system transformation in Sub-Saharan Africa under different development pathways, using long-term changes in caloric availability, adjusted protein density, the balance between animal and plant proteins, and the diversification of animal protein sources as the empirical basis for this assessment. **Methods:** The analysis is based on data from the Food and Agriculture Organization of the United Nations for the period 1961–2023, covering Nigeria, the Republic of the Congo, the Democratic Republic of the Congo, Kenya, South Africa and Ethiopia. A longitudinal approach was applied where data were available, complemented by a recent comparative analysis (2010–2023). Derived indicators include total protein supply, adjusted protein density, the share of animal and plant proteins and the Herfindahl–Hirschman index (HHI) to assess the diversification of animal protein sources. **Results:** Findings show that increases in caloric availability are not consistently associated with improvements in adjusted protein density or nutritional quality. Significant differences are observed across countries in protein supply levels, structural composition, and diversification patterns. Results reveal heterogeneous trajectories of food systems, from diversified to structurally constrained or highly concentrated systems. **Conclusions:** Beyond caloric availability, the structure and quality of the food supply are essential for assessing food system performance. Quantitative gains alone do not ensure improved nutritional or sustainable outcomes. The study highlights the importance of considering protein supply structure, diversification and nutritional efficiency in the context of SDG 2 (Zero Hunger) and broader food system transformation.

## 1. Introduction

Global food systems are currently under unprecedented pressure, driven by the complex interplay of population growth, climate change, natural resource degradation and accelerating socio-economic transformations. In this context, ensuring food security can no longer be analyzed exclusively from the perspective of quantitative food availability, but must be integrated into a broader framework that includes nutritional, sustainability and equity dimensions [1,2].

The literature increasingly highlights the interdependencies between food security, nutrition and the sustainability of food systems. Thus, producing enough quantity of food does not automatically guarantee access to healthy and balanced diets, and increasing agricultural production can, under certain conditions, generate negative effects on the environment and on the health of the population. In this sense, the concept of “sustainable diets” has been defined as those dietary patterns that have a reduced environmental impact, contribute to food and nutritional security, and are culturally and economically acceptable [3,4].

Within this conceptual framework, a crucial distinction emerges between two paradigms: “more food” and “better food”. The former focuses on increasing the quantity of food production and availability, while the latter emphasizes nutritional quality, food diversity and the sustainability of production and consumption systems. This transition from quantity to quality represents one of the main challenges of contemporary food systems [4,5,6].

Traditionally, food availability has been assessed through quantitative indicators, particularly average per capita caloric supply. Although this indicator remains essential for assessing the risk of undernutrition and overall food availability, it has significant limitations in assessing the nutritional quality of diets [4,5,6].

Increased caloric availability does not necessarily reflect improved supply of essential nutrients, particularly high-quality protein. In many contexts, increased caloric supply is associated with increased reliance on carbohydrate-rich foods that are low in protein or micronutrients, which can lead to multiple forms of malnutrition, including nutritional deficiencies and overnutrition [6,7,8].

In this context, a more nuanced approach to food systems analysis is needed, going beyond simple calorie quantification and integrating structural dimensions of the diet. Two concepts are particularly relevant in this regard: protein supply structure and protein density. Protein structure reflects the distribution of protein sources between animal and plant components, as well as the internal diversity of animal-based proteins. Protein density, expressed as the ratio of total protein to caloric supply, provides a synthetic measure of the nutritional efficiency of the food system.

These two paradigms and two concepts are operationalized throughout the analytical framework of this study as follows. The “more food” paradigm is captured empirically through caloric availability (kcal/capita/day), while the “better food” paradigm is captured through the structural and qualitative dimensions of the protein supply. These two structural dimensions are, in turn, measured through the two concepts introduced above: protein supply structure, operationalized as the share of animal- and plant-based proteins and the Herfindahl–Hirschman concentration index of animal protein sources (Section 2.3, Equations (7) and (8)); and protein density, operationalized as the ratio of total protein to caloric supply, both in its original (Equation (3)) and loss-adjusted (Equation (5)) forms. Protein density thus functions as the analytical bridge between the two paradigms, translating the quantitative dimension of food availability (“more food”) into a measure of nutritional efficiency relevant to the “better food” paradigm, while protein supply structure provides a complementary, compositional perspective on the same transition. This operationalization underpins the structure of Section 3, where RQ1 addresses the “more food” paradigm (Section 3.1), RQ2 addresses protein density as the bridge to the “better food” paradigm (Section 3.2), and RQ3–RQ4 address protein supply structure (Section 3.3, Section 3.4 and Section 3.5).

Food systems analysis therefore requires a shift in perspective from an exclusively calorie-focused approach to one that integrates qualitative and structural dimensions of nutrition. This perspective is summarized in the concept of “Beyond Calories,” which emphasizes the need to simultaneously assess the quantity and quality of available food.

Sub-Saharan Africa is one of the most dynamic and at the same time vulnerable regions in terms of food security. The region is characterized by a significant diversity of food systems, reflected in major differences between countries in terms of the level of economic development, the structure of agricultural production and food consumption patterns [7,8,9].

Despite economic progress in recent decades, many countries in the region continue to face high levels of food insecurity and malnutrition. This situation is often associated with structural factors such as political instability, climate variability, limited agricultural infrastructure, and unequal access to resources. At the same time, some countries are experiencing an increase in food availability, raising questions about the nutritional quality of this increase [9,10].

A defining feature of the diet in many countries in Sub-Saharan Africa is the high proportion of plant-based proteins, particularly from cereals and legumes, at the expense of animal-based proteins. This dietary pattern reflects both economic constraints and cultural and resource availability factors [9,10].

This study examines six countries representative of the region’s diversity: Nigeria, Republic of the Congo (Congo), Democratic Republic of the Congo (DR Congo), Kenya, South Africa, and Ethiopia.

They provide a suitable comparative framework for analyzing different trajectories of food system development in the region.

Although the existing literature extensively addresses issues related to food security and nutrition in Sub-Saharan Africa, most studies focus either on quantitative indicators, such as caloric availability, or on specific indicators of malnutrition, without integrating a structural perspective on the composition of diets [10,11,12].

There is a lack of studies that simultaneously analyze long-term developments in caloric availability and protein supply structure, using integrated indicators such as protein density. Also, existing approaches rarely investigate the internal diversity of animal-based protein sources and its implications for the sustainability of food systems [10,11,12,13].

This gap is more relevant in the current context, where food and nutrition policies must simultaneously address the objectives of food security, public health, and sustainability. Without an integrated understanding of the relationship between the quantity and quality of available food, there is a risk of promoting strategies that increase food availability but do not lead to real improvements in nutritional status [13,14,15].

Therefore, an analytical approach is needed that goes beyond the quantitative paradigm and integrates the structural dimensions of nutrition, thus providing a more solid basis for formulating sustainable food policies.

The aim of this study is to examine what drives and constrains the transformation of food systems in Sub-Saharan Africa under different development pathways, using the long-term evolution of caloric availability, protein supply structure and protein density as the empirical basis for this assessment. Rather than treating increased caloric availability as a uniform proxy for nutritional progress, the study explicitly accounts for the fact that countries have followed markedly different caloric growth trajectories over the period 1961–2023 and examines how these differences relate to structural and qualitative outcomes in protein supply. In this context, the study aims to answer the following research questions:

RQ1. How has caloric availability evolved in the countries analyzed, and to what extent has this evolution been accompanied by changes in the level of total protein supply?

RQ2. Does increasing caloric availability lead to improvements in protein density and structural quality of the food supply?

RQ3. How has the structure of the protein supply, the ratio of animal- and plant-based proteins, and the internal composition of animal-based proteins changed?

RQ4. What differences can be observed between recent food system profiles, and what do they reveal about the diversity of food system development trajectories in Sub-Saharan Africa?

While RQ1–RQ4 provide the descriptive and diagnostic basis of the analysis, the overarching aim of the study is captured by the following synthesis question:

RQ5. What are the drivers and constraints of food system transformation across different development pathways in Sub-Saharan Africa, and to what extent does the magnitude of caloric growth account for the divergence observed in nutritional outcomes?

## 2. Materials and Methods

### 2.1. Data Sources

The analysis presented in this study is based on a combination of quantitative data and bibliographic sources relevant to the field of food security and sustainable food systems.

The primary source of the quantitative data is the FAOSTAT Food Balance Sheets, compiled by the Statistics Division of the Food and Agriculture Organization of the United Nations. Data were accessed through the Our World in Data (OWID) platform, version accessed on 1 February 2026 [16,17,18]. OWID was used instead of direct extraction from FAOSTAT because it reconciles the discontinuity between FAOSTAT’s earlier Food Balance Sheets methodology (covering 1961–2013) and the updated methodology introduced from 2010 onwards, providing a single continuous time series for the full 1961–2023 period required for the longitudinal analysis presented in this study.

Data quality control involved two main procedures. First, missing values were handled by excluding DR Congo from the long-term longitudinal analysis (1961–2023), as time series data for this country are only available from 2010 onwards; DR Congo was included exclusively in the recent comparative analysis (2010–2023), as described in Section 2.2. Second, the time series for each country and indicator were visually inspected for improbable values or sudden discontinuities and cross-checked with official FAO reports, where necessary, to identify potential data quality issues before calculating derived indicators.

Quantitative data were taken from the Our World in Data (OWID) platform, which integrates and harmonizes statistical information from international sources, in particular from the databases of the Food and Agriculture Organization of the United Nations (FAO). The dataset used covers the period 1961–2023 and includes the following variables:

- total food availability expressed in kilocalories per capita per day (kcal/capita/day) [16],
- animal- and plant-based protein availability (g/capita/day) [17], and
- detailed structure of animal-based proteins, broken down by main categories: fish and seafood, poultry, pork, beef and buffalo, sheep and goat meat, other meats, eggs and dairy products [18].

In parallel, to substantiate the theoretical framework and interpret the results, relevant bibliographic sources identified in academic databases such as Web of Science and Google Scholar were consulted, as well as grey literature, including reports from international organizations (FAO, WHO, World Bank) and documents from national institutions in the field [1,4].

The selection process of bibliographic sources was based on the use of combinations of keywords such as food security, sustainable diets, protein consumption, dietary patterns, nutrition transition, Sub-Saharan Africa, and food systems. Articles published in indexed scientific journals, comparative studies, and recent institutional reports relevant to the analysis of the relationship between food availability, nutritional structure, and the food systems’ sustainability were prioritized.

### 2.2. Country Selection

The selection of the six countries in Sub-Saharan Africa—Nigeria, DR Congo, Congo, Kenya, South Africa, and Ethiopia—was made deliberately, based on the following criteria:

(i) economic diversity, reflected in significant differences in development levels;
(ii) nutritional diversity, highlighted by different food supply structures; and
(iii) regional representativeness, by including countries from West, Central, East, and Southern Africa.

An important methodological aspect is the simultaneous inclusion of Congo and DR Congo, two countries that are politically and economically distinct but geographically close. This choice allows to highlight significant differences between seemingly similar regional food systems.

Regarding data availability, the time series for DR Congo is limited to the period 2010–2023, which required the exclusive use of this country in the recent comparative analysis and its exclusion from the long-term longitudinal analysis.

### 2.3. Calculated Indicators

Based on the initial variables extracted from the Our World in Data database, a series of derived indicators was calculated, designed to capture both the quantitative and structural dimensions of the food supply.

First, the total amount of available protein was determined as the sum of animal- and plant-based proteins, according to the relationship:

(1) $$Protein_{total}=Protein_{animal-based}+Protein_{plant-based}$$

Based on this variable, structural indicators, such as the share of animal- and plant-based proteins in total proteins, as well as the ratio between them (animal/plant ratio), were calculated, used to highlight the balance between protein sources.

The formula for calculating the variations from 1961 to 2023 was:

(2) $$\Delta \%=\frac{value_{2023}-value_{1961}}{value_{1961}}\times 100$$

To assess the nutritional quality of food supply, the protein density indicator was used, defined as the ratio between the total amount of protein and the total caloric supply, expressed in g/1000 kcal:

(3) $${Protein\;density}_{total}=\frac{Prot{ein}_{total}\;g/day/capita}{Energy\;kcal/day/capita}\times 1000$$

Similarly, the density of animal-based protein was also calculated as an indicator of high-quality protein supply in relation to available energy.

In addition, to assess the impact of food losses and waste on energy availability, a caloric adjustment scenario was constructed, based on FAO‘s estimates of the average level of food losses in Sub-Saharan Africa [19]. Thus, caloric availability was adjusted by applying a −23% reduction factor:

(4) Adjusted Energy=Energy×(1−0.23)

The adjustment was applied exclusively to caloric availability (kcal/capita/day), as aggregate estimates of food losses are mainly available for the energy dimension. Protein availability was not adjusted, given the absence of comparable and disaggregated coefficients for losses related to different protein sources (animal- and plant-based).

A uniform, region-wide loss factor was applied rather than country-specific or commodity-specific rates because internationally comparable, country-level food loss estimates are only available through FAO’s SDG Indicator 12.3.1a monitoring framework, which uses 2015 as its baseline year and therefore does not provide historical coverage for the 1961–2023 period examined in this study. Consequently, the −23% adjustment should be interpreted as a regional average approximation applied consistently across the full time series, rather than as a precise, country- or commodity-specific correction.

Based on this adjustment, protein density was recalculated using adjusted energy, according to Formula (5), which integrates both the original protein density definition (Formula (3)) and the caloric adjustment factor (Formula (4)).

(5) $${Protein\;density}_{ajusted\;for\;food\;losses}=\frac{Protein_{total}}{Adjusted\;Energy}\times 1000$$

This adjusted indicator provides a closer approximation of the effective nutritional availability of protein by accounting for potential losses occurring along the food supply chain.

The internal structure of animal-based proteins was analyzed by determining the share of each category (fish, poultry, pork, beef, sheep, and goat meat, eggs and dairy) in total animal-based proteins. This approach allows highlighting the differences between protein sources and their implications for the sustainability of diets.

For the comparative graphical representation of the analyzed indicators (caloric availability, adjusted protein density, and protein source structure), a min–max normalization procedure was applied, in order to bring them onto a common scale. Normalization was performed based on the values of the indicators for 2023, to allow comparison of recent food system profiles between the analyzed countries.

Normalization was performed according to the relationship:

(6) $$\mathrm{Normalized}\;\mathrm{value}=\frac{x-min}{max-min}\times 100$$

where

x—the initial value of the indicator.

min and max—minimum and maximum values in the analyzed data set.

This transformation was used for comparative visualization, to allow the comparability of indicators expressed in different units without affecting the real values used in the analysis and interpretation of the results.

To assess the degree of diversification of animal-based protein sources, the Herfindahl–Hirschman concentration index (HHI) was used, calculated according to the formula [20]:

(7) $$HHI=\sum_{i=1}^{n}s_{i}^{2}$$

where

s_i_—the share of each protein category in total animal-based proteins

calculated as follows:

(8) $$s_{i}=\frac{Quantity\;from\;source\;of\;animal-based\;protein}{Total\;quantity\;of\;animal-based\;protein}\times 100$$

Higher HHI values indicate a higher concentration of protein sources, while lower values reflect a higher diversification of the diet [20]:

- Low HHI (~0.15–0.20) → diversified diet.
- High HHI (>0.30) → concentration (dependence on a few sources).

This approach allows the assessment of differences between the theoretical availability of food and the actual potential level of energy supply, as well as their impact on the nutritional structure of the diet.

### 2.4. Analytical Approach

The analysis was carried out by combining a longitudinal with a comparative and structural perspective. The longitudinal analysis focused on the evolution of indicators over the period 1961–2023 for countries for which complete data series are available, namely Nigeria, Congo, Kenya, South Africa and Ethiopia. This allowed the identification of long-term trends in caloric availability and the structure of protein supply.

The recent comparative analysis was carried out for the period 2010–2023 and included all six countries analyzed, including the DR Congo. This stage allowed the highlighting contemporary differences in food system profiles.

The structural analysis focused on both the distribution of proteins between animal- and plant-based sources and the internal composition of animal-based proteins. For this purpose, the previously described derived indicators, including protein density and concentration index, were used.

Various visual methods were used to represent the results, adapted to the type of analysis, including line graphs for temporal evolutions, stacked diagrams for protein structure, synthetic comparative representations, and heatmap graphs for recent food system profiles.

### 2.5. Statistical Analysis

To complement the descriptive analysis and examine the relationship between caloric availability, total protein supply, and protein density, a non-parametric correlation analysis was performed using Kendall’s Tau coefficient (τ), along with associated *p*-values to assess statistical significance. The analysis was conducted separately for each country, examining the relationships between the time series of caloric availability (c) and protein density (pd), as well as between total protein supply (tp) and protein density (pd).

Kendall’s Tau coefficient was selected due to its robustness in capturing monotonic relationships between variables, without assuming linearity or normal distribution. This is particularly relevant in the context of long-term structural trends in food systems, where relationships between variables may be nonlinear and influenced by structural changes.

Although the underlying dataset covers the entire period 1961–2023, correlation analysis was performed on selected time points to ensure consistency and comparability in interpreting long-term trends. The statistical analysis was performed using SAS Studio 3.8, and significance was assessed at a threshold of *p* < 0.05.

## 3. Results

This section is structured around the four research questions (RQ1–RQ4) and progressively aims to analyze the evolution of caloric and protein availability, to assess protein density as an indicator of nutritional quality, identify structural changes in protein supply (animal vs. plant), and to compare recent profiles of food systems in the countries analyzed.

Through these analytical directions, the results allow an integrated examination of the relationship between the amount of available food and the nutritional structure of the diet, as well as of differences between food development trajectories.

### 3.1. Evolution of Caloric and Protein Availability (RQ1)

The evolution of caloric availability in the countries analyzed, as well as the extent to which it is accompanied by changes in the total level of protein supply, is examined based on the data summarized in Table 1. Total protein supply was calculated according to Formula (1), while percentage variations over the period 1961–2023 were computed using Formula (2), as defined in Section 2.3.

Caloric availability shows divergent trajectories across the analyzed countries over the period 1961–2023. Significant increases are observed in Ethiopia (+46.37%) and Nigeria (+26.46%), while Congo records a moderate increase (+9.07%). In contrast, Kenya exhibits a noticeable decline (−8.88%), and South Africa shows a slight decrease (−4.47%). For DR Congo, the long-term analysis is constrained by data availability, which only begins after 2010.

These heterogeneous trajectories are consistent with previous studies highlighting the uneven nature of food system development and nutritional transitions in Sub-Saharan Africa, shaped by differences in agricultural systems, economic development, and access to food [21,22].

The evolution of protein availability does not uniformly follow the dynamics of caloric availability, revealing important differences between quantitative changes in food supply and its nutritional composition. In Ethiopia, the substantial increase in caloric availability (+46.37%) is accompanied by a strong rise in protein supply (+38.00%), indicating a relatively coherent improvement in both energy and nutritional supply. A similar, though more moderate, pattern is observed in Nigeria (+26.46% kcal; +24.68% protein), suggesting a proportional evolution between the two indicators.

In contrast, Congo presents a markedly different trajectory. While caloric availability increases only slightly (+9.07%), protein availability rises sharply (+75.27%), indicating a significant structural shift in the composition of the diet, with a growing relative importance of protein supply.

A different pattern is observed in Kenya, where both caloric availability (−8.88%) and protein supply (−23.54%) decline over the long term, suggesting a deterioration in both the quantity and the nutritional dimension of food supply.

South Africa exhibits a relatively stable profile, characterized by a slight decrease in caloric availability (−4.47%) combined with a moderate increase in protein supply (+6.12%), indicating limited but positive changes in the nutritional structure of the food system.

For DR Congo, the available data for the period 2010–2023 indicate relatively low levels of protein supply (27.72 g/capita/day in 2023), but the absence of long-term data prevents a comprehensive assessment of historical trends.

To address the possibility that the magnitude of caloric growth itself explains differences in nutritional outcomes, the five countries included in the longitudinal analysis were grouped according to the direction of their caloric growth trajectory over 1961–2023 (Table 2).

Countries with positive caloric growth (Ethiopia, Nigeria, Congo) do not display a consistent pattern in adjusted protein density: Ethiopia and Nigeria show slight declines (−5.73% and −1.40%, respectively), while Congo shows a substantial increase (+60.71%). Similarly, countries with declining caloric availability (Kenya, South Africa) diverge sharply, with Kenya showing the steepest decline in protein density observed in the sample (−16.07%) and South Africa showing a moderate improvement (+11.08%). This grouped comparison indicates that the rate of caloric growth does not, by itself, systematically predict the direction of change in nutritional quality—reinforcing the need to examine country-specific structural and economic drivers, as discussed in Section 4.4, rather than treating caloric growth as a uniform trajectory across the region. Given the small number of countries in each group (*n* = 3 and *n* = 2, respectively), this comparison is intended as an illustrative, exploratory grouping rather than a formal statistical test of between-group differences.

**Figure 1:**
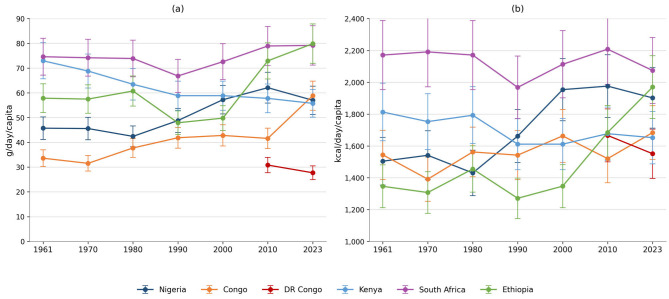
Long-term trends in caloric availability (**a**) and total protein supply (**b**), in selected countries in Sub-Saharan Africa (1961–2023). Error bars represent an illustrative ±10% margin applied to each data point, reflecting the general order of magnitude of measurement uncertainty commonly associated with national-level Food Balance Sheet estimates. They do not represent an empirically derived statistical error and are unrelated to the food-loss adjustment factor described in Section 2.3. Source: Original by authors.

Figure 1 illustrates the long-term trajectories of caloric availability and protein supply, highlighting both the heterogeneity across countries and the uneven relationship between energy supply and nutritional composition.

### 3.2. Beyond Calories—Protein Density (RQ2)

To assess the extent to which increased food availability is associated with improvements in nutritional quality, the analysis was extended using an adjusted protein density indicator. This indicator was calculated based on adjusted energy availability, according to Formula (5), which integrates the original definition of protein density (Formula (3)) with the caloric adjustment factor that takes into account food losses (Formula (4)), as defined in Section 2.3.

This adjusted indicator provides an analytical approximation of the potential nutritional availability of protein by taking into account the potential 23% losses that occur along the food supply chain in Africa [19].

The results highlight an uneven relationship between caloric availability and protein density, suggesting distinct food system trajectories. Although caloric availability has increased in most of the countries analyzed, this evolution is not systematically accompanied by an improvement in protein density, indicating a predominantly quantitative, rather than structural, transformation of the food supply (Table 3).

A first pattern is the relative stability of adjusted protein density, as observed in Nigeria. Despite a substantial increase in caloric availability, adjusted protein density shows only minor fluctuations over time, with a slight decrease from 39.49 to 38.94 g/1000 kcal. This suggests that the increase in food availability has not translated into a significant improvement in the nutritional efficiency of the diet.

A second pattern is that of a strong improvement in adjusted protein density, illustrated by Congo. In this case, the indicator increases significantly, from 28.26 to 45.42 g/1000 kcal, indicating a substantial structural change in the composition of dietary supply towards a higher protein content in relation to the effective energy availability. This development is consistent with the strong increase in protein supply identified in Section 3.1 and suggests a significant qualitative transformation of the diet.

A third pattern is represented by the decrease in adjusted protein density, as observed in Kenya. The decrease from 52.25 to 43.86 g/1000 kcal indicates a reduction in the relative protein content of the diet, suggesting a shift towards more energy-dense and low-protein food sources. This trend reflects a deterioration in the nutritional composition of the diet, despite relatively moderate changes in caloric availability.

South Africa shows a moderate increase in adjusted protein density, from 44.64 to 49.58 g/1000 kcal, indicating a gradual improvement in the nutritional composition of the diet. This suggests that, even in the context of relatively stable caloric availability, the composition of dietary supply has shifted towards a higher protein profile.

In Ethiopia, adjusted protein density remains relatively high throughout the period under review, although it is characterized by fluctuations and a slight overall decrease (from 55.84 to 52.64 g/1000 kcal). This suggests that despite significant increases in both caloric and protein availability, the balance between energy and protein supply has not improved proportionately.

**Figure 2:**
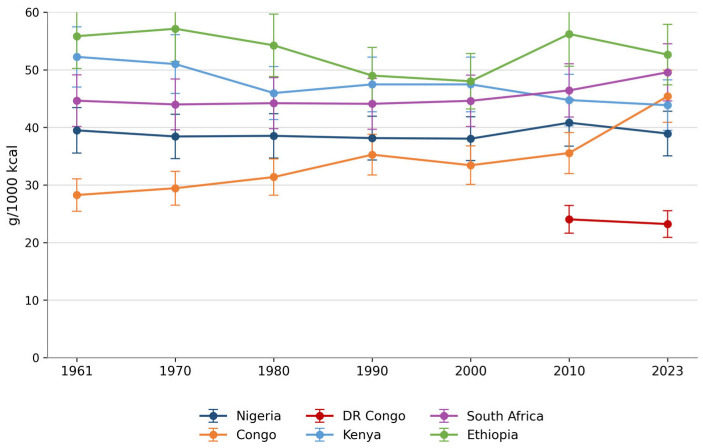
Long-term trends in protein density in selected Sub-Saharan African countries (1961–2023) (g/1000 kcal). Error bars represent an illustrative ±10% margin applied to each data point, reflecting the general order of magnitude of measurement uncertainty commonly associated with national-level Food Balance Sheet estimates. They do not represent an empirically derived statistical error and are unrelated to the food-loss adjustment factor described in Section 2.3. Source: Original by authors.

In the case of DR Congo, the analysis is limited to the period 2010–2023. Available data indicate relatively low and slightly decreasing values of adjusted protein density, from 24.03 to 23.21 g/1000 kcal, suggesting a food system with structural constraints and limited nutritional efficiency. Figure 2 illustrates the long-term trends in protein density.

Overall, the results indicate that increases in caloric availability do not systematically lead to improvements in adjusted protein density. Therefore, the relationship between quantity and quality of food supply is mediated by the structural composition of the diet, rather than simply the absolute level of food availability. This finding aligns with studies showing that increases in caloric availability do not necessarily correspond to improvements in dietary quality or protein adequacy [23,24].

The dynamics summarized by percentage change (Δ% 1961–2023) further highlight the lack of a consistent relationship between caloric availability and adjusted protein density, as well as significant divergences across countries. The results range from a substantial increase in Congo (+60.71%) to notable decreases in Kenya (−16.07%) and Ethiopia (−5.73%), while Nigeria shows relative stability (−1.40%) and South Africa records a moderate increase (+11.08%).

The magnitude and direction of these variations highlight the uneven nature of food system transitions in the region and reinforce the central argument of this study: quantitative progress in food systems does not necessarily imply an improvement in nutritional quality.

Overall, the results indicate that the relationship between the quantity and quality of food supplies depends on the internal structure of food systems and cannot be inferred solely from the evolution of caloric indicators. Thus, the analysis provides a clear answer to RQ2 and supports the need to integrate structural indicators, such as adjusted protein density, into the assessment of food system performance.

These differences suggest that food systems evolve along multiple, nonlinear trajectories, shaped by structural and compositional changes, rather than just caloric expansion.

#### Relationship Between Caloric Availability, Protein Supply, and Protein Density

As a sensitivity analysis complementing the descriptive trends presented in Section 3.2, a correlation analysis was conducted between caloric availability, total protein supply, and adjusted protein density (i.e., the loss-adjusted indicator defined in Section 2.3), using the Kendall Tau coefficient (Table 4). This analysis is exploratory in nature and is intended to test the robustness of the observed patterns rather than to establish the primary basis for the study’s conclusions, which rest on the descriptive longitudinal and structural analyzes presented throughout Section 3.

The results indicate the absence of statistically significant correlations between caloric availability and protein density in most of the countries analyzed, including Nigeria and Ethiopia (*p* > 0.05). This suggests that increases in energy availability are not systematically associated with improvements in the nutritional efficiency of the food supply.

In contrast, moderate positive correlations were observed between total protein supply and protein density in Congo and South Africa, indicating that, in these cases, quantitative increases in protein supply are partly reflected in improvements in nutritional structure.

**Figure 3:**
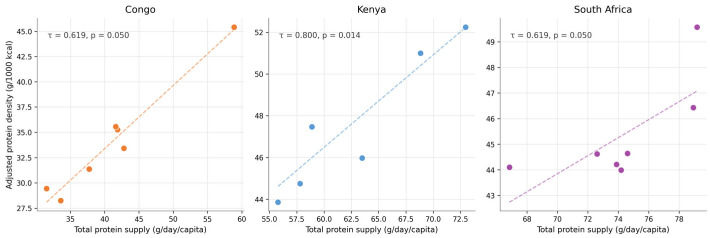
Scatter plot matrix illustrating the relationship between total protein supply and protein density in certain countries where moderate or statistically significant correlations were observed (Congo, Kenya, South Africa). Each point represents paired values of total protein supply and protein density. The distribution of the points reflects the strength of the association between the variables, and the dashed line represents the linear trend between the two variables, shown for illustrative purposes only. The graphical representation reflects the relative positioning of the values, rather than their chronological sequence. Source: Original by authors using SAS Studio 3.8.

A distinct pattern is observed in Kenya, where both caloric availability and protein density show a decreasing trend, accompanied by a strong and statistically significant correlation (τ = 0.800, *p* = 0.014), suggesting a structurally consistent but negative trajectory (Figure 3).

The graphical representation is intended as a complementary visualization of these relationships and should be interpreted as illustrative, given the number of observations included in the correlation analysis. Overall, these findings provide empirical support for the central argument of this study: that increasing food quantity does not necessarily translate into improvements in nutritional quality, as captured by protein density. The descriptive trends illustrated in Figure 1 and Figure 2 are complemented by the Kendall Tau correlation analysis presented in Table 4, which provides an exploratory statistical assessment of the relationships between caloric availability, protein supply, and protein density.

### 3.3. Structural Changes in Protein Supply (RQ3)

To further explore the structural dimension of food supply, the analysis was extended to the composition of proteins according to their source, namely animal-based and plant-based proteins. This approach allows the assessment not only of the total amount of protein available, but also of the structural quality of the diet.

The results highlight significant differences between countries in the ratio of animal-based and plant-based proteins, as well as divergent developments over time (Table 5).

A first pattern identified is that of the persistent dominance of plant-based proteins, characteristic of Nigeria and Ethiopia. In these cases, the structure of protein supply remains strongly oriented towards plant sources, despite increases in the total amount of protein. The long-term dynamics confirm this trend: in Nigeria, plant-based proteins increase by +23.21%, while animal-based proteins register a higher relative increase (+37.37%), suggesting a modest shift in the structure of protein supply. In Ethiopia, the dependence on plant sources is increasing, reflected by a +56.45% increase in plant-based proteins, concomitant with a decrease in animal-based proteins (−38.40%), which indicates a polarization of the food structure. This predominance of plant-based proteins is consistent with previous studies describing the persistence of cereal and legume-based dietary patterns in several countries in Sub-Saharan Africa, often associated with limited access to animal-based foods [25,26].

A second pattern is represented by the transition towards a higher share of animal-based proteins, illustrated by Congo. In this country, animal-based proteins show an exceptional increase (+150.18%), while plant-based proteins increase moderately (+22.36%). This evolution indicates a major structural transformation of the diet, reflecting a significant increase in the role of animal-based protein sources. Similar transitions towards higher consumption of animal-based foods have been reported in some rapidly transforming African food systems, particularly in contexts of urbanization characterized by changing dietary patterns and increased consumption of poultry [27,28].

A third pattern is that of relative stability of protein structure, observed in Kenya. Both animal (−14.62%) and plant-based proteins (−26.25%) show long-term decreases, without major changes in the ratio between them. This evolution suggests the absence of a clear structural transition and reflects fluctuations in food availability, rather than systemic transformations.

In contrast, South Africa presents a distinct profile, characterized by the consolidation of the role of animal-based proteins. Their increase (+46.08%), concomitant with a reduction in plant-based proteins (−13.99%), indicates a reconfiguration of the food structure towards a higher share of animal-based protein, suggesting increased dietary complexity and diversification.

**Figure 4:**
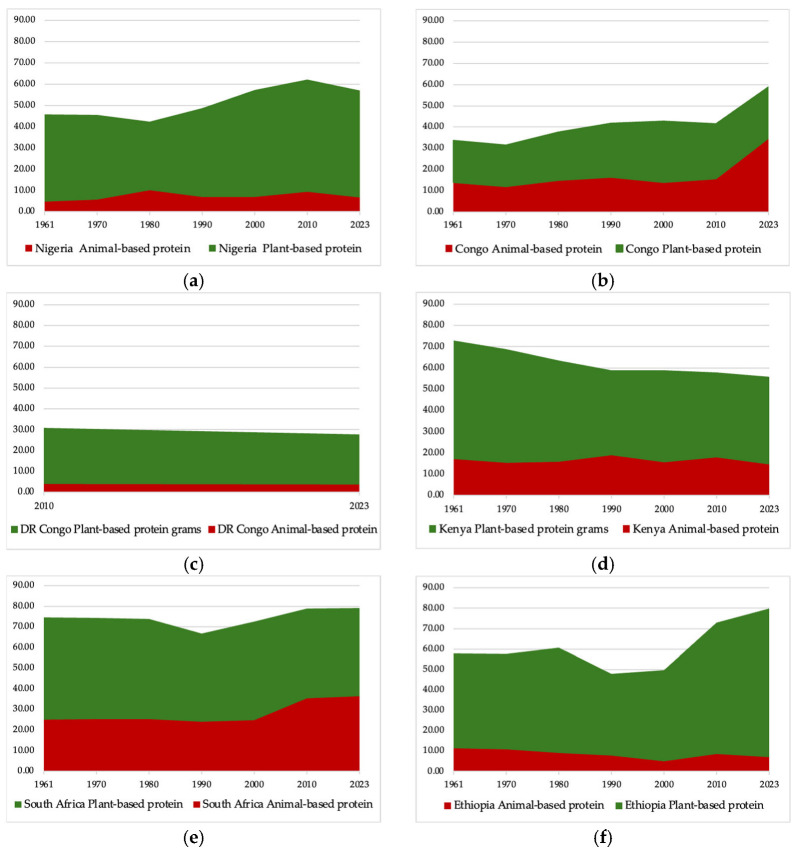
Long-term trends in the structure of animal-based vs. plant-based protein supply in selected Sub-Saharan African countries (**a**) Nigeria; (**b**) Congo; (**c**) DR Congo; (**d**) Kenya; (**e**) South Africa; (**f**) Ethiopia (g/day/capita). Source: Original by authors.

In the case of DR Congo, the analysis is limited by the availability of data only for the period 2010–2023. During this period, the structure of protein supply is dominated by plant-based proteins, while the supply of animal-based proteins remains low. In the absence of a complete time series, the long-term dynamics cannot be assessed, but the recent profile suggests the persistence of significant structural constraints (Figure 4).

Overall, the results indicate that food transitions in the region do not follow a single pattern but reflect divergent trajectories, from increasing reliance on plant-based proteins to accelerating increases in animal-based protein supply. Thus, the structure of protein supply becomes a key element in understanding the nutritional quality of food systems. These findings are consistent with recent literature that highlights the heterogeneity of food system transitions in Sub-Saharan Africa and the coexistence of multiple food trajectories shaped by economic, cultural, and structural factors [29,30]. Similar structural vulnerabilities, including limited diversification of protein sources and reduced access to animal-based foods, have also been reported in previous studies addressing food insecurity and nutritional fragility in Central African food systems [31,32]. Recent studies also highlight that food insecurity and nutritional fragility in Sub-Saharan Africa are shaped by complex structural inequalities, limited diversification of food systems, and uneven transitions between plant-based and animal-based protein sources, with important implications for sustainability and resilience [33,34].

These structural differences help explain the observed variations in adjusted protein density (Section 3.2), highlighting the role of protein source composition in shaping the nutritional quality of food systems.

The analysis, therefore, answers RQ3 and highlights that improving protein supply depends not only on increasing total protein supply but also on the balance and diversification of protein sources.

### 3.4. Recent Food Systems Profiles (RQ4)

To complement the longitudinal analysis and integrate the case of DR Congo, this section examines recent food system profiles, focusing on the most recent year available (2023). This approach allows for direct comparison across countries and highlights contemporary differences in the structure and quality of food supplies.

The analysis is based on the set of key indicators (caloric availability, adjusted protein density, and protein supply structure), the values of which are presented in Table 4. To ensure comparability between indicators expressed in different units, a min–max normalization procedure was applied, according to formula (6). The normalization was performed using values corresponding to the year 2023, allowing for a consistent comparison of recent food system profiles across the countries analyzed (Table 6).

**Figure 5:**
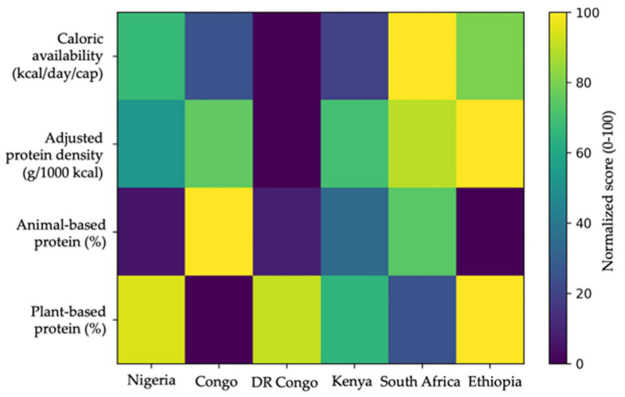
Recent profiles of food systems in selected Sub-Saharan African countries (2023), based on caloric availability, adjusted protein density, and protein supply structure. Indicators are normalized (0–100) using min–max scaling. Source: Authors’ representation.

The results indicate the existence of distinct profiles, defined by specific combinations of caloric availability, adjusted protein density and protein supply structure (Figure 5).

A first profile is represented by relatively diversified food systems with a significant share of animal protein, illustrated by South Africa. This pattern is characterized by high caloric availability, a relatively high adjusted protein density and a balanced structure of protein sources, suggesting a more complex and diversified food system.

A second profile is that of food systems in transition, exemplified by Congo. In this case, the strong increase in animal protein supply is accompanied by a substantial increase in adjusted protein density, indicating a significant improvement in the nutritional efficiency of the food supply. However, this transformation is associated with a relatively high concentration of protein sources, suggesting that structural improvements coexist with limited diversification.

A third profile is represented by plant-dominated food systems, illustrated by Nigeria and Ethiopia. In these countries, the structure of protein supply remains strongly biased towards plant sources, despite increasing food availability. In Ethiopia, this pattern is particularly pronounced, reflecting a high reliance on plant proteins, even in the presence of relatively high values of adjusted protein density. Kenya represents an intermediate profile, characterized by moderate caloric availability, decreasing adjusted protein density, and a relatively concentrated structure of protein sources. This configuration suggests limited progress in improving nutritional quality and diversifying the food system.

The DR Congo is distinguished by a profile characterized by low levels of caloric availability and low adjusted protein density, combined with a protein structure dominated by plant sources. Although the analysis is limited to the period 2010–2023, the available data indicate a structurally constrained and nutritionally fragile food system.

A comparative analysis of these profiles highlights that food systems in Sub-Saharan Africa do not converge towards a single development model but follow divergent trajectories shaped by structural, economic, and resource-related factors. The analysis therefore provides a clear answer to RQ4 and highlights the need for differentiated approaches in assessing and designing food policies.

### 3.5. Diversifying Animal Protein Sources

To assess the degree of diversification of animal-based protein sources, the Herfindahl–Hirschman concentration index (HHI) was used, calculated according to Formula (7), based on the share of each protein category in total animal-based protein, as defined by Formula (8) (Table 7).

The results highlight significant differences between countries in terms of the degree of diversification of protein sources, suggesting the existence of distinct food structures (Table 8).

A first pattern is represented by diversified food systems, characterized by low HHI values, illustrated by Nigeria (0.1675). In this case, animal-based protein supply is relatively evenly distributed across multiple categories, with a diversified protein structure, which may contribute to greater resilience. At the opposite end, Kenya (0.3848) and Congo (0.3405) show high levels of concentration, suggesting a strong dependence on a limited number of protein sources. In the case of Congo, this concentration is mainly driven by the strong growth of poultry-based protein, while in Kenya, the structure is dominated by dairy products. Ethiopia (0.3084) also falls into the category of relatively concentrated food systems, reflecting limited diversification of animal-based protein sources and a dependence on a narrow set of traditional products. South Africa (0.2786) presents an intermediate profile, characterized by greater diversification compared to the previously mentioned countries, but without reaching the level of balance observed in Nigeria. In the case of the DR Congo (0.2138), the index values suggest a moderate degree of diversification, but interpretation should be made with caution, given the limited availability of data only for the period 2010–2023.

The analysis based on the HHI index highlights that the diversification of animal-based protein sources represents an essential dimension of the quality of food systems, complementary to the quantitative and structural indicators analyzed previously.

### 3.6. Synthesis of Food Systems Development Trajectories

The integrated analysis of quantitative, structural, and diversification indicators allows the identification of distinct food system development trajectories in the countries analyzed. These trajectories reflect specific combinations of caloric availability, adjusted protein density, protein supply structure, and the degree of diversification of animal sources, while providing relevant indications on the degree of sustainability of these food systems. They were derived through the integrated interpretation of the indicators presented in Table 1, Table 2, Table 3, Table 4, Table 5, Table 6, Table 7 and Table 8 and Figure 1, Figure 2, Figure 3, Figure 4 and Figure 5, in particular, adjusted protein density, protein supply structure, caloric availability, and diversification of animal protein sources (HHI).

The results highlight the existence of four main types of trajectories.

A first type is represented by diversified and relatively balanced food systems, illustrated by Nigeria. This profile is characterized by a relatively stable adjusted protein density, a structure dominated by plant proteins, and a high degree of diversification of animal protein sources (low HHI). This combination suggests a relatively robust food system, with a favorable potential for sustainability, by balancing sources and reducing dependence on a single food category.

A second type is that of food systems in structural transition, illustrated by Congo. This profile is defined by an accelerated increase in animal protein, accompanied by a significant increase in adjusted protein density and a high level of source concentration (high HHI). This trajectory indicates a continuous but uneven food transition, in which improvements in nutritional quality coexist with limited diversification of protein sources.

A third type is represented by concentrated food systems, dependent on a limited number of protein sources, as illustrated by Kenya and Ethiopia. High HHI values indicate low diversification and a supply structure dominated by specific sources. In Kenya, this is associated with a decrease in adjusted protein density, while in Ethiopia, adjusted protein density remains relatively high, despite a strong dependence on plant sources. This trajectory reflects structural vulnerabilities and low resilience, with implications for the long-term sustainability of food systems.

A fourth type is that of relatively diversified food systems with an emphasis on animal-based proteins, exemplified by South Africa. This profile is characterized by high animal protein supply, an increase in adjusted protein density, and a diversified diet structure, suggesting a higher level of food system complexity. However, the implications for sustainability need to be considered in a nuanced manner, given the greater resource impact of animal protein production.

The DR Congo stands out with a particular trajectory, characterized by structural constraints and limited data availability. The recent profile indicates a high reliance on plant sources, a low adjusted protein density, and relatively low caloric availability, suggesting a fragile food system with significant limitations in terms of access and diversification, essential elements for sustainability.

**Figure 6:**
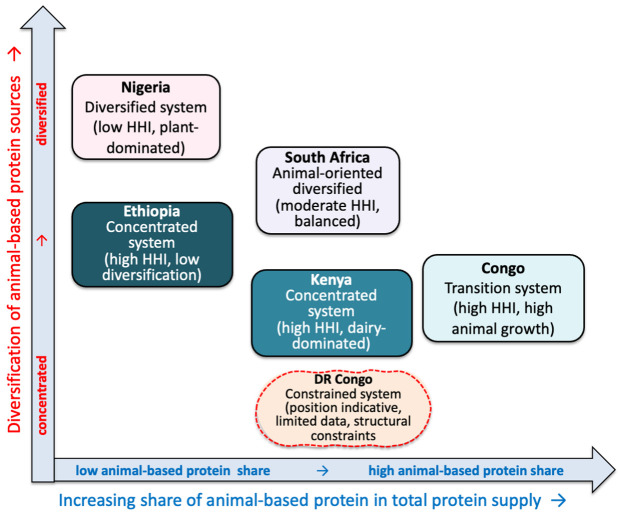
Typology of food system development trajectories in selected Sub-Saharan African countries, based on the share of animal-based protein in total protein supply and the degree of diversification of animal-based protein sources. Source: Original by authors.

The typology illustrated in Figure 6 was derived through the integrated interpretation of the indicators analyzed throughout the study, in particular adjusted protein density, caloric availability, protein supply structure, and diversification of animal protein sources (HHI) (Figure 6).

The results highlight that food systems in Sub-Saharan Africa are not evolving towards a convergent pattern, but rather are following divergent trajectories, with different implications for sustainability. This typology provides a useful conceptual framework for assessing food system performance and supporting country-specific policies.

These differences in diversification help explain the observed variations in adjusted protein density (Section 3.2), highlighting the importance of protein source diversity for the nutritional performance of food systems.

### 3.7. Focused Perspective—Congo vs. DR Congo

The comparative perspective focusing on the differences between Congo and DR Congo highlights the contrasts between two food systems located in the same geographical region, which share similar natural resources and common cultural roots. In this context, the relative comparability of the initial conditions enhances the relevance of the differences observed in the structure and performance of these food systems.

Although the two countries have some structural similarities, the results indicate clearly divergent trajectories in terms of food availability, the structure of the protein supply, the adjusted protein density, and the degree of diversification of animal protein sources.

Congo is characterized by a significant increase in the supply of animal protein, especially from poultry, leading to a structural reconfiguration of the diet. This evolution is accompanied by a strong increase in the adjusted protein density, indicating an improvement in the nutritional efficiency of the food supply. However, it is also associated with a high concentration of protein sources (HHI = 0.3405), reflecting a strong dependence on a limited number of food categories.

In contrast, the DR Congo presents a food system characterized by low levels of caloric availability, low adjusted protein density, and low supply of animal protein. Although the HHI value (0.2138) suggests a moderate degree of diversification, this should be interpreted in the context of low absolute levels of consumption and limited data availability. In this case, the apparent diversification does not reflect a balanced or resilient food system, but rather the absence of a dominant source of protein.

The comparison of the two cases highlights that the transition to higher levels of animal protein consumption does not automatically translate into a sustainable or balanced food system. While Congo illustrates a trajectory of nutritional improvement accompanied by structural focus, DR Congo remains constrained by systemic limitations that affect food access, diversification, and overall nutritional efficiency. Overall, this focused analysis demonstrates that food systems performance must be assessed through an integrated approach that simultaneously considers quantity, structure, diversification, and nutritional efficiency, alongside the broader country-specific institutional and economic context.

## 4. Discussion

### 4.1. Beyond Calories—The Limits of Food Progress in the Context of SDG 2—Zero Hunger of the UN’s 2030 Agenda

The results obtained should be interpreted in the broader context of the global commitments set out in the UN 2030 Agenda for Sustainable Development, in particular Sustainable Development Goal 2 (SDG 2—Zero Hunger), which aims to end hunger, all forms of malnutrition, and ensure universal access to safe, nutritious, and sufficient diets. With less than four years remaining until the 2030 deadline, empirical evidence indicates that these goals are still far from being achieved, particularly in Sub-Saharan Africa [19,35,36].

The results of this study provide not only descriptive but also empirical evidence to support this limitation. Correlation analysis conducted between caloric availability, total protein supply, and protein density shows that, in most cases, increases in energy availability are not significantly associated with improvements in nutritional quality. The absence of statistically significant correlations in countries such as Nigeria and Ethiopia, along with moderate associations in Congo and South Africa, indicates that the relationship between food quantity and nutritional composition is neither linear nor systematic. This suggests that improvements in food availability may not systematically translate into better nutritional outcomes, even in contexts of sustained quantitative growth.

Statistically, the correlation results presented in Table 3 should be interpreted with caution, given the limited number of paired observations available per country. However, the observed pattern is informative: caloric availability was not significantly correlated with protein density in Nigeria, Ethiopia, and South Africa (*p* > 0.05), indicating no statistically detectable monotonic relationship between the two variables over the period analyzed. In contrast, Kenya showed a strong and statistically significant correlation between total protein intake and protein density (τ = 0.800, *p* = 0.014), reflecting a coherent, structurally consistent—albeit negative—trajectory in which both indicators decreased together. Congo and South Africa showed correlations close to the conventional significance threshold (*p* = 0.050), suggesting an emerging, although not yet fully established, association between total protein intake and nutritional density. Taken together, these results indicate that where a statistically significant relationship was detected, it did not necessarily correspond to nutritional improvement, strengthening the argument that increases in food quantity are not systematically associated with better nutritional outcomes.

In this context, the findings of the present study provide a critical perspective on how progress in food systems should be assessed. These findings reinforce the broader interpretation of the results, indicating that increases in caloric availability are not systematically associated with improvements in nutritional quality, as reflected in protein density. This disconnect between the quantitative and qualitative dimensions of food supply highlights a fundamental limitation of traditional approaches that assess food system performance primarily through energy-based indicators [37,38].

Furthermore, the analysis suggests that achieving SDG 2.1, which requires universal access to sufficient and nutritious food, cannot be assessed solely through quantitative measures. Even in cases where caloric availability increases, this does not necessarily translate into improved food quality or reduced nutritional vulnerability. This finding reinforces the multidimensional nature of food security, which must integrate not only availability and access, but also the nutritional quality and structural composition of diets [39,40]. This suggests that food system progress should be assessed not only in terms of availability, but also in terms of structural nutritional composition. However, it should be noted that the analysis is based on food availability data, which does not accurately reflect actual consumption patterns, as differences in access, income distribution, and intra-household allocation can influence the extent to which available food translates into efficient nutritional supply.

Similarly, SDG 2.2, focused on eradicating all forms of malnutrition, is challenged by the high variability observed in adjusted protein density and the structure of food supplies across countries. The results indicate that improving nutritional outcomes cannot be achieved solely by increasing food volume but requires specific interventions aimed at improving dietary composition, protein quality, and diversifying food sources [41,42].

The findings also provide important insights for SDG 2.4, which promotes sustainable and resilient food systems. The analysis shows that increases in animal protein supply are not necessarily associated with improved sustainability, especially when such increases are accompanied by a high concentration of protein sources. This lack of diversification may reduce system resilience and increase vulnerability to external shocks, highlighting the importance of dietary diversity as a key component of sustainable food systems [43,44].

Another key contribution of this study is the identification of heterogeneous food system trajectories. The significant differences observed across countries indicate that there is no single path to food system development in Sub-Saharan Africa. While some countries experience quantitative growth without structural improvements, others show contrasting dynamics, including stagnation or decline in certain dimensions. These findings highlight the need for food policies to be context-specific and tailored to the structural characteristics of each food system, rather than relying on uniform models [45,46]. Overall, the results highlight a fundamental discrepancy between the ambitions of SDG 2 and the empirical realities of the food systems under review. While increases in food availability remain necessary, they are not sufficient to ensure progress towards ending hunger and malnutrition. Without a structural transformation of food systems—focused on diversification, nutritional quality, and resilience—progress is likely to remain partial and insufficient to address the complexity of today’s global challenges [47,48].

### 4.2. Implications for Sustainable Diets

The results of this study have direct implications for the current debate on sustainable diets, particularly in the context of food system transformation in Sub-Saharan Africa. Recent literature highlights that sustainable diets must simultaneously meet nutritional, economic, and environmental criteria, being accessible, diverse, and compatible with ecosystem boundaries [4,43,49].

The study results reinforce these insights, showing that the sustainability of diets cannot be assessed solely by the amount of nutrients available. The results indicate that increases in total protein supply are not consistently associated with improvements in protein density, highlighting the importance of structural composition and balance between protein sources.

A first key implication concerns the role of protein diversity. Analysis of the structure of protein supply and the degree of concentration of animal sources shows that, in many cases, food systems are characterized by low diversification. This lack of diversity is associated not only with structural vulnerabilities but also with lower levels of adjusted protein density, indicating reduced nutritional efficiency. These findings reinforce the idea that dietary diversity is a key determinant of both nutritional health and food system resilience, especially in contexts marked by climatic and economic instability [50,51,52]. This is further supported by the HHI results, which show that a higher concentration of protein sources does not necessarily correspond to improved nutritional outcomes and may, in some cases, reflect structural constraints rather than adequate nutrition.

At the same time, the results highlight a complex and nonlinear relationship between animal protein consumption and dietary sustainability. In several of the countries analyzed, increases in animal protein supply are associated with improvements in total protein availability, but not necessarily with higher adjusted protein density or greater diversification of sources. This finding suggests that increased animal protein consumption cannot be considered in itself a sufficient indicator of nutritional progress. This is consistent with the literature, which emphasizes both the nutritional benefits of animal protein and its environmental implications [3,53,54].

Therefore, the results of this study do not support a simplistic interpretation in which increased animal protein consumption automatically reflects improved diet quality. Instead, they indicate that the structure and diversification of protein sources play a more important role than the absolute level of consumption. This perspective aligns with recent approaches that promote balanced diets based on an appropriate mix of plant and animal protein sources [13,55].

A third important implication concerns the role of alternative protein sources, particularly those of plant origin and traditional or underutilized crops. In several of the countries analyzed, plant proteins remain the dominant component of the diet; however, this reliance is not always associated with high nutritional diversity or quality. The findings suggest that improving diet quality requires not only maintaining plant-based dominance, where appropriate, but also increasing diversity within plant protein sources. This is supported by the literature, which highlights the potential of local crops and alternative proteins to contribute to both food security and sustainability [56,57,58].

Furthermore, ongoing transformations of food systems in Sub-Saharan Africa, including increased consumption of processed foods and changes in dietary patterns, may have significant implications for long-term nutritional quality and sustainability [59,60]. In this context, the study highlights the need for an integrated approach that considers not only food availability but also supply chain structure, access to diverse foods, and consumption behavior.

The findings therefore indicate that the transition to sustainable diets cannot be achieved through simple increases in food availability or unidirectional changes in consumption patterns. Instead, a structural reconfiguration of food systems is needed, focusing on diversifying protein sources, improving nutritional efficiency (as reflected by adjusted protein density), and adapting dietary patterns to the specific socio-economic and ecological context of each country.

### 4.3. Implications for Public Policies

The results of the study highlight the need for a fundamental reconfiguration of food systems policies, particularly in Sub-Saharan Africa, where the heterogeneity of food system trajectories is significantly more pronounced than aggregate approaches suggest. The differences observed across countries indicate that uniform or standardized policies are insufficient to address the structural complexity of food systems in the region. This finding is consistent with recent literature that emphasizes the importance of adaptive governance and context-specific policymaking [61,62,63].

A first key implication concerns the need to differentiate intervention strategies according to the structural and nutritional profile of each country. The results of this study show that increases in food availability are not always accompanied by improvements in adjusted protein density or diversification of protein sources. Therefore, in systems characterized by quantitative growth without structural improvements, policy interventions should prioritize diversifying food supplies and improving nutritional quality, rather than focusing exclusively on increasing production. In contrast, in systems with higher levels of diversification, policy priorities may shift towards stability, accessibility, and strengthening supply chain efficiency. This suggests that policymaking needs to be aligned with specific food system trajectories, rather than relying on generalized development patterns.

A second important implication concerns the role of investments in infrastructure, agricultural research, and technological development, in line with SDG 2. While increasing productive capacity remains essential, the results indicate that such investments should also support diversification of production and promote food sources with higher nutritional value. In particular, improving adjusted protein density requires not only increasing production but also improving the structural composition of food systems [21,64].

At the same time, the findings highlight the importance of strengthening food system resilience to climate, economic, and geopolitical shocks. The observed variability in adjusted protein density and protein supply structure indicates that many food systems in Sub-Saharan Africa remain vulnerable to external shocks. These results suggest that resilience cannot be achieved by increasing production alone but requires diversification, flexibility, and improved adaptive capacity within food supply chains [65,66].

Another relevant issue concerns the functioning of food markets and access to information, in line with SDG 2. Market inefficiencies and price volatility can significantly affect access to food, even in contexts of apparent food availability. The literature indicates that improving market transparency and access to information is essential to reduce volatility and strengthen food security outcomes [67,68].

Finally, the results of this study highlight the importance of the social dimension of food systems, particularly for smallholder farmers, women, and vulnerable populations, in line with SDG 2.3. Increasing their productivity and income is not only an economic objective, but also a structural condition for improving access to diverse and nutritionally adequate diets. This finding supports the need to integrate social inclusion and equity into food policymaking [52,69].

The policy implications indicate that transforming food systems in Sub-Saharan Africa requires moving beyond one-size-fits-all approaches. Instead, an integrated policy framework is needed that combines increased production with structural diversification, improved nutritional efficiency, and increased resilience. Without such a multidimensional and context-sensitive approach, progress towards SDG 2 is likely to remain uneven and insufficient.

### 4.4. Diversity of Food Trajectories

One of the most relevant findings of this study is the diversity of food system development trajectories in Sub-Saharan Africa. The comparative analysis demonstrates that food systems do not follow a single or convergent pattern, but rather reflect several distinct trajectories, shaped by specific combinations of economic, agricultural, institutional, and cultural factors.

These divergent trajectories can be partially explained by structural and economic drivers well documented in the nutrition transition literature. Rising income and urbanization tend to increase demand for animal-based protein, following the pattern described by Popkin’s nutrition transition framework and the “livestock revolution” identified by Delgado et al. (2001) [70], which is consistent with the accelerating animal-protein trajectories observed in Congo and South Africa. Trade liberalization and access to relatively cheap imported poultry and dairy products have similarly supported animal-protein growth in some West and Southern African economies. In contrast, agroecological constraints, such as the presence of tsetse fly belts limiting cattle husbandry across large parts of the Congo Basin, together with a predominance of subsistence agriculture based on staple crops (e.g., cassava, plantain, legumes), help explain the persistence of plant-based protein reliance in DR Congo, Nigeria, and Ethiopia. Similar heterogeneity has been documented outside Sub-Saharan Africa: rapid income growth in parts of Southeast Asia and Latin America has been associated with sharp increases in animal-protein and processed-food consumption, whereas in parts of South Asia, cultural dietary preferences and land constraints have sustained high reliance on plant-based protein despite comparable economic growth, a pattern structurally similar to that observed here in Nigeria and Ethiopia [70,71].

This result challenges the assumption, present in some parts of the literature, that food systems evolve according to generalized patterns of nutritional transition applicable at regional or global scales. While classical concepts of “nutritional transition” provide a useful framework for understanding general changes in nutrition [7,72], the findings of this study show that such models do not adequately capture the structural heterogeneity observed across countries.

In contrast to linear interpretations, the analysis reveals divergent configurations in which caloric availability, adjusted protein density, protein supply structure and diversification of animal protein sources (HHI) evolve independently or even in opposite directions. This indicates that quantitative progress does not necessarily translate into improvements in nutritional quality or system resilience.

These findings suggest that food systems in Sub-Saharan Africa are better understood as adaptive systems, characterized by multiple, context-dependent pathways, rather than a single development trajectory. This perspective aligns with recent approaches in the literature that emphasize the plurality of food system transformations and the absence of a universal development model [43,73].

Furthermore, the results highlight the role of structural factors, including agricultural systems, supply chain organization and local consumption patterns, in shaping these trajectories. At the same time, broader processes such as urbanization, market integration, and the expansion of processed food consumption introduce additional layers of complexity, generating both diversification and new forms of nutritional imbalance [59,74].

A key implication of these findings is that policy interventions cannot be designed based on standardized models or mechanically transferred across countries. Instead, effective strategies need to be tailored to the specific trajectory of each food system, taking into account its structural characteristics, level of diversification, and nutritional performance.

The diversity of food trajectories identified in this study provides a strong argument for moving beyond simplified, linear perspectives on food system transformation. Rather than converging towards a single model, food systems evolve along multiple paths, each shaped by context-specific constraints and opportunities. Recognizing this diversity is essential for developing more effective, context-sensitive, and sustainable food policies.

### 4.5. Study Limitations and Shortcomings

The results of this study should be interpreted taking into account several methodological limitations, mainly related to the nature of the data used and the constraints associated with long-term comparative analysis.

First, the indicator of caloric availability (kcal/day/capita) reflects the amount of food available at the end of the supply chain, rather than actual individual consumption. Therefore, it may overestimate actual energy supply, as it does not take into account food losses and waste at the consumer level. This limitation is well documented in the literature and is inherent in the use of Food Balance Sheet data, which provide an aggregated perspective on food availability but not on actual consumption patterns [6,10].

To partially address this limitation, the analysis incorporated an exploratory scenario of adjusting for caloric availability, based on FAO estimates that food losses in Sub-Saharan Africa may reach approximately 23% along the supply chain, from post-harvest stages to the retail level [1,35]. This adjustment was used to derive the adjusted protein density indicator and should not be interpreted as an empirical correction of the data, but rather as an analytical tool designed to assess the sensitivity of nutritional indicators to variations in actual food availability.

An additional limitation concerns the uniform nature of this adjustment. Although country-specific or commodity-specific food loss rates would provide a more precise correction, such data are only available through FAO’s SDG Indicator 12.3.1a monitoring framework from 2015 onwards, and therefore do not cover the 1961–2023 period analyzed in this study. As a result, the –23% factor represents a regional average approximation rather than a country-specific estimate, and the adjusted protein density indicator should accordingly be interpreted as a sensitivity-analysis tool, complementary to the descriptive findings presented in Section 3, rather than as the primary empirical basis for the study’s conclusions.

A related but distinct methodological clarification concerns the error bars displayed in Figure 1 and Figure 2. These represent an illustrative ±10% margin applied uniformly to each data point, reflecting the general order of magnitude of measurement uncertainty commonly associated with national-level Food Balance Sheet estimates, rather than an empirically derived statistical error calculated from the underlying data. This illustrative margin is conceptually independent from, and should not be confused with, the −23% food-loss adjustment factor discussed above, which applies specifically to the caloric adjustment scenario described in Section 2.3 and is unrelated to the visual representation of uncertainty in Figure 1 and Figure 2.

Second, the limited availability of data for the DR Congo constrained the longitudinal analysis to the period 2010–2023 for this country. Therefore, comparisons involving the DR Congo should be interpreted with caution, as they are not fully comparable, from a temporal perspective, with the complete time series available for the other countries. However, the inclusion of DR Congo remains relevant for capturing the diversity of recent food system profiles in the region.

Third, the indicators used to characterize the structure of protein supply, including the Herfindahl–Hirschman Index (HHI), only provide an approximation of diversification. These indicators do not capture important dimensions such as protein quality, nutrient bioavailability or the intra-population distribution of consumption. Therefore, the analysis remains limited to the level of aggregate food supply and does not allow for a detailed assessment of individual nutritional outcomes. Furthermore, the normalization procedure applied for visualization purposes (Figure 5), based on min–max scaling, introduces a degree of abstraction, as it transforms indicators into relative scores. While this approach facilitates comparability between indicators, it does not reflect absolute magnitudes and should be interpreted accordingly.

Fourth, another limitation concerns the level of detail available for protein sources. While animal-based proteins are disaggregated into multiple categories, plant-based proteins are only available as an aggregate measure. This limits the ability to assess the internal diversity of plant-based protein sources, which can play a significant role in determining dietary quality.

Fifth, the correlation analysis presented in Section “Relationship Between Caloric Availability, Protein Supply, and Protein Density” is based on a small number of paired observations per country (*n* = 7, corresponding to the years selected at approximately decade intervals), which limits its statistical power and the robustness of the Kendall Tau estimates. Consequently, the correlation results should be regarded as exploratory and hypothesis-generating rather than confirmatory, and non-significant results should not be interpreted as evidence of the absence of a relationship. Furthermore, because the analysis relies on discrete time points rather than the full annual series, it does not account for autocorrelation or potential structural breaks within the underlying 1961–2023 series. Future research could address these constraints through time-series econometric approaches—such as autoregressive or error-correction models that explicitly account for autocorrelation and structural break tests (e.g., Chow or Bai–Perron tests)—applied to the complete annual series, which would allow for a more robust assessment of the relationship between caloric availability, protein supply, and nutritional quality over time.

Despite these limitations, the study provides a robust analytical framework for examining the relationship between food availability, supply structure, diversification, and nutritional efficiency, as captured by adjusted protein density. By integrating quantitative and structural indicators in a long-term comparative perspective, the analysis contributes to a more nuanced understanding of food system dynamics and highlights the existence of divergent development trajectories in Sub-Saharan Africa. The limitations identified do not invalidate the results, but rather define the scope within which they should be interpreted and point to important directions for future research, including integrating consumption data, assessing protein quality and bioavailability, and refining indicators that capture the nutritional and environmental dimensions of food systems.

## 5. Conclusions

This study examined the drivers and constraints of food system transformation in Sub-Saharan Africa under different development pathways, using the long-term evolution of caloric availability, protein supply structure, and adjusted protein density as the empirical basis for this assessment. Rather than treating caloric growth as a uniform trajectory across the region, the analysis explicitly incorporated the magnitude of caloric change as an analytical dimension, showing that it does not, by itself, account for the divergent nutritional outcomes observed across countries. The results demonstrate that increases in caloric availability are not systematically associated with improvements in nutritional quality. The analysis of adjusted protein density reveals the absence of a consistent relationship between food quantity and nutritional efficiency, confirming that quantitative progress does not automatically translate into nutritional progress.

Furthermore, the findings highlight that structural transformations in protein supply follow divergent patterns across countries. Differences in the balance between animal and plant proteins, as well as in the degree of diversification of animal sources, reflect the existence of multiple food system trajectories, rather than a single development path.

From this perspective, the study highlights that the structure of protein supply represents a key dimension for understanding food system transformation. Beyond the total volume of food, the composition and diversification of protein sources directly influence nutritional quality, system resilience, and sustainability outcomes, as Africa faces both the climate crisis and food insecurity, two interconnected realities that profoundly affect the future of the continent [75,76,77]. The findings therefore clearly show that, beyond calories, the structure and quality of the food supply are essential.

The results have important implications for the implementation of the UN 2030 Agenda, in particular SDG 2. Although this goal aims to end hunger and all forms of malnutrition, the analysis shows that progress in food availability is insufficient. Achieving SDG 2 requires a multidimensional approach that integrates not only availability but also nutritional quality, diversification, and resilience of food systems. Moreover, these dimensions are closely interconnected with other SDGs, including poverty reduction (SDG 1), health and well-being (SDG 3), responsible consumption and production (SDG 12), and climate action (SDG 13).

The study also highlights significant implications for public policy. Strategies focused solely on increasing food production and availability are insufficient to ensure food and nutrition security. Instead, policies need to adopt a differentiated and context-specific approach, targeting not only production levels but also the structural composition of food systems, diversifying protein sources, and improving nutritional efficiency.

In terms of future research, the findings highlight the need to further develop indicators that capture protein quality, nutrient bioavailability, and the environmental impact of food systems. Furthermore, integrating supply data with consumption information would allow for a more comprehensive understanding of the relationship between food availability and nutritional outcomes.

Overall, the study demonstrates that progress towards the goals of the UN 2030 Agenda cannot be assessed solely by increasing food availability. Without a structural transformation of food systems—particularly in terms of protein composition and diversification—there is a risk that quantitative progress will not translate into significant improvements in food security and sustainability. This underlines the need for a shift from quantity-oriented approaches to structurally informed and nutrition-sensitive food systems strategies.

## Figures and Tables

**Table 1:** Evolution of caloric and protein availability in the countries analyzed (1961–2023), using values selected at intervals of approximately one decade [16,17].

Year		Country	Nigeria	Congo	DR Congo	Kenya	South Africa	Ethiopia
	Calories/Total Protein							
1961	Calories kcal/cap/day		1504	1544	—	1813	2171	1346
	Total protein g/day/capita		45.74	33.60	—	72.96	74.61	57.87
1970	Calories kcal/cap/day		1541	1391	—	1753	2191	1307
	Total protein g/day/capita		45.60	31.53	—	68.83	74.19	57.50
1980	Calories kcal/cap/day		1430	1562	—	1793	2171	1455
	Total protein g/day/capita		42.43	37.75	—	63.48	73.89	60.77
1990	Calories kcal/cap/day		1662	1542	—	1611	1968	1271
	Total protein g/day/capita		48.83	41.88	—	58.89	66.84	47.93
2000	Calories kcal/cap/day		1954	1663	—	1611	2113	1347
	Total protein g/day/capita		57.25	42.81	—	58.89	72.60	49.81
2010	Calories kcal/cap/day		1976	1520	1666	1677	2208	1685
	Total protein g/day/capita		62.11	41.61	30.83	57.79	78.94	72.95
2023	Calories kcal/cap/day		1902	1684	1551	1652	2074	1970
	Total protein g/day/capita		57.03	58.89	27.72	55.78	79.18	79.87
Δ% 1961–2023	Calories kcal/cap/day		26.46%	9.07%	—	−8.88%	−4.47%	46.37%
	Total protein g/day/capita		24.68%	75.27%	—	−23.54%	6.12%	38.00%

Note: Values are taken from the annual Our World in Data series [16,17] and are presented at approximately 10-year intervals (1961, 1970, 1980, 1990, 2000, 2010, and 2023) to facilitate long-term comparability. For DR Congo, data is only available after 2010. This applies to all data presented below.

**Table 2:** Grouping of countries by caloric growth trajectory (1961–2023) and corresponding change in adjusted protein density.

Growth Group	Country	Δ% Calories (1961–2023)	Δ% Adjusted Protein Density (1961–2023)
Caloric growth	Ethiopia	46.37%	−5.73%
Caloric growth	Nigeria	26.46%	−1.40%
Caloric growth	Congo	9.07%	60.71%
Caloric decline	Kenya	−8.88%	−16.07%
Caloric decline	South Africa	−4.47%	11.08%

**Table 3:** Evolution of protein density in countries selected from Sub-Saharan Africa (1961–2023), using values selected at approximately one-decade intervals (g/1000 kcal).

	Country	Nigeria	Congo	DR Congo	Kenya	South Africa	Ethiopia
Year							
1961		39.49	28.26	—	52.25	44.64	55.84
1970		38.43	29.44	—	51	43.99	57.13
1980		38.54	31.38	—	45.97	44.21	54.25
1990		38.15	35.27	—	47.47	44.1	49
2000		38.05	33.43	—	47.47	44.62	48.02
2010		40.82	35.56	24.03	44.75	46.43	56.22
2023		38.94	45.42	23.21	43.86	49.58	52.64
Δ% 1961–2023		−1.40%	60.71%	—	−16.07%	11.08%	−5.73%

**Table 4:** Kendall Tau correlation coefficients (τ) and corresponding *p*-values for the relationships between caloric availability (c), total protein supply (tp), and protein density (pd) in selected Sub-Saharan African countries.

Country	τ (c vs. pd)	*p*-Value (c vs. pd)	τ (tp vs. pd)	*p*-Value (tp vs. pd)
Nigeria	−0.047	0.880	0.047	0.880
Congo	0.238	0.452	0.619	0.050
Kenya	0.300	0.356	0.800	0.014
South Africa	0.000	1.000	0.619	0.050
Ethiopia	−0.047	0.880	0.142	0.652

Note: DR Congo was not included in this analysis due to the limited availability of comparable long-term data.

**Table 5:** Evolution of the structure of animal-based vs. plant-based protein supply in selected countries in Sub-Saharan Africa (g/day/capita) [17].

Country		Year	1961	1970	1980	1990	2000	2010	2023	Δ% 1961–2023
	Protein Source (g/Day/Capita)									
Nigeria	Animal-based protein		4.79	5.61	10.06	7.03	7.00	9.37	6.58	37.37%
	Plant-based protein		40.95	39.99	32.37	41.80	50.25	52.74	50.45	23.21%
Congo	Animal-based protein		13.91	11.91	14.77	16.24	13.86	15.46	34.80	150.18%
	Plant-based protein		19.69	19.62	22.98	25.64	28.95	26.15	24.09	22.36%
DR Congo	Animal-based protein		—	—	—	—	—	3.96	3.65	—
	Plant-based protein		—	—	—	—	—	26.87	24.07	—
Kenya	Animal-based protein		17.03	15.25	15.85	18.83	15.48	17.87	14.54	−14.62%
	Plant-based protein		55.93	53.58	47.63	40.06	43.41	39.92	41.24	−26.25%
South Africa	Animal-based protein		24.99	25.35	25.36	24.03	24.71	35.29	36.51	46.08%
	Plant-based protein		49.62	48.84	48.53	42.81	47.89	43.65	42.67	−13.99%
Ethiopia	Animal-based protein		11.25	10.83	8.96	7.91	4.89	8.60	6.93	−38.40%
	Plant-based protein		46.62	46.67	51.81	40.02	44.92	64.35	72.94	56.45%

**Table 6:** Normalized values (0–100) of caloric availability, adjusted protein density, and protein supply structure in selected Sub-Saharan African countries (2023).

Country	Caloric Availability (kcal/Day/Cap)	Adjusted Protein Density (g/1000 kcal)	Protein Share Animal-Based (%)	Protein Share Plant-Based (%)
Nigeria	67.11	53.43	5.67	94.33
Congo	25.43	75.48	100	0
DR Congo	0	0	8.91	91.09
Kenya	19.31	70.18	34.5	65.5
South Africa	100	89.61	74.23	25.77
Ethiopia	80.11	100	0	100

**Table 7:** Structural composition and evolution of animal-based protein supply by source (g/day/capita) in selected Sub-Saharan African countries [18].

	Source of Animal-Based Protein (g/Day/Capita)	1961	1970	1980	1990	2000	2010	2023
Nigeria	Fish and seafood	1.10	1.09	3.64	2.32	1.98	3.55	1.93
	Poultry	0.20	0.31	0.56	0.55	0.39	0.66	0.64
	Pork	0.15	0.16	0.17	0.35	0.40	0.54	0.63
	Beef and buffalo	1.38	1.41	2.15	0.84	0.88	0.77	0.59
	Sheep and goats	0.11	0.20	0.46	0.65	1.03	1.32	0.90
	Other meat protein	0.85	0.88	0.67	0.47	0.47	0.48	0.40
	Eggs	0.43	0.51	0.71	0.92	0.84	1.01	0.82
	Dairy	0.32	0.78	1.33	0.58	0.57	0.69	0.41
Congo	Fish and seafood	8.17	6.07	8.37	10.34	6.00	4.77	6.24
	Poultry	0.33	0.27	0.66	1.62	1.69	3.15	16.54
	Pork	0.36	0.41	0.22	0.43	0.74	0.39	2.87
	Beef and buffalo	1.11	1.63	1.55	0.60	0.64	1.05	1.14
	Sheep and goats	0.09	0.08	0.13	0.20	0.14	0.18	0.12
	Other meat protein	3.19	2.89	2.75	2.31	2.49	4.33	3.99
	Eggs	0.09	0.07	0.13	0.10	0.15	0.14	0.06
	Dairy	0.46	0.37	0.78	0.29	1.42	1.11	0.56
DR Congo	Fish and seafood	-	-	-	-	-	1.43	0.99
	Poultry	-	-	-	-	-	0.53	0.35
	Pork	-	-	-	-	-	0.27	0.23
	Beef and buffalo	-	-	-	-	-	0.11	0.13
	Sheep and goats	-	-	-	-	-	0.16	0.10
	Other meat protein	-	-	-	-	-	1.19	0.83
	Eggs	-	-	-	-	-	0.04	0.02
	Dairy	-	-	-	-	-	0.13	0.50
Kenya	Fish and seafood	0.57	1.02	0.90	2.23	1.79	0.85	0.73
	Poultry	0.30	0.46	0.68	0.27	0.18	0.29	0.77
	Pork	0.07	0.02	0.05	0.06	0.10	0.11	0.26
	Beef and buffalo	5.31	4.51	4.85	3.77	3.39	4.64	1.79
	Sheep and goats	1.50	0.89	0.87	0.97	0.85	0.99	0.89
	Other meat protein	0.75	0.63	0.56	0.54	0.44	1.07	0.81
	Eggs	0.25	0.23	0.27	0.44	0.47	0.57	0.39
	Dairy	6.55	6.21	6.23	9.25	6.87	8.26	8.28
South Africa	Fish and seafood	2.71	3.27	2.62	2.73	1.81	1.70	1.53
	Poultry	0.80	1.90	3.01	5.40	7.59	14.77	15.33
	Pork	0.86	0.93	0.79	0.85	0.65	1.63	2.09
	Beef and buffalo	8.59	6.83	7.79	6.26	5.37	7.10	6.54
	Sheep and goats	2.59	2.89	1.96	1.49	1.57	1.42	1.06
	Other meat protein	0.01	0.03	0.04	0.13	0.11	0.32	0.44
	Eggs	0.82	1.19	1.41	1.21	1.61	1.86	1.83
	Dairy	6.36	6.32	5.73	4.21	4.17	4.53	4.51
Ethiopia	Fish and seafood	0.02	0.07	0.03	0.03	0.07	0.06	0.13
	Poultry	0.50	0.52	0.54	0.46	0.19	0.29	0.23
	Pork	0.01	0.01	0.01	0.01	0.01	0.01	0.01
	Beef and buffalo	3.39	3.44	2.26	1.98	1.75	1.94	1.40
	Sheep and goats	1.89	1.60	1.33	1.07	0.33	0.74	0.97
	Other meat protein	1.12	1.14	1.25	1.19	0.46	0.65	0.50
	Eggs	0.54	0.51	0.47	0.38	0.11	0.11	0.09
	Dairy	2.21	2.12	2.01	1.91	1.37	4.21	3.06

**Table 8:** Herfindahl–Hirschman Index (HHI) of animal-based protein source diversification in selected Sub-Saharan African countries (2023).

Country	Nigeria	Congo	DR Congo	Kenya	South Africa	Ethiopia
HHI	0.1675	0.3405	0.2138	0.3848	0.2786	0.3084

## Data Availability

The original contributions presented in this study are included in the article material. Further inquiries can be directed to the corresponding author. The statistical analysis, including Kendall’s Tau correlation, was performed using SAS Studio 3.8, available at https://documentation.sas.com/doc/en/sasstudiocdc/3.8/webeditorcdc/webeditorref/titlepage.htm (accessed on 5 January 2026).
